# Photovoltaic Properties and Ultrafast Plasmon Relaxation Dynamics of Diamond-Like Carbon Nanocomposite Films with Embedded Ag Nanoparticles

**DOI:** 10.1186/s11671-017-2065-1

**Published:** 2017-04-20

**Authors:** Šarūnas Meškinis, Domantas Peckus, Andrius Vasiliauskas, Arvydas Čiegis, Rimantas Gudaitis, Tomas Tamulevičius, Iryna Yaremchuk, Sigitas Tamulevičius

**Affiliations:** 1 Institute of Materials Science, Kaunas University of Technology, K. Baršausko St. 59, Kaunas, LT-51423 Lithuania; 2 Department of Photonics, Lviv Polytechnic National University, S. Bandera Str. 12, Lviv, 79013 Ukraine

**Keywords:** Localized surface plasmon resonance, Silver nanoparticles, Photovoltaic properties, Diamond-like carbon, Electron-phonon interaction, Charge transfer, Hot electrons, Magnetron sputtering, Nanocomposites

## Abstract

Ultrafast relaxation dynamics of diamond-like carbon (DLC) films with embedded Ag nanoparticles (DLC:Ag) and photovoltaic properties of heterojunctions consisting of DLC:Ag and crystalline silicon (DLC:Ag/Si) were investigated by means of transient absorption (TAS) spectroscopy and photovoltaic measurements. The heterojunctions using both p type and n type silicon were studied. It was found that TAS spectra of DLC:Ag films were dependent on the used excitation wavelength. At wavelengths where Ag nanoparticles absorbed light most intensively, only DLC signal was registered. This result is in good accordance with an increase of the DLC:Ag/Si heterojunction short circuit current and open circuit voltage with the excitation wavelength in the photovoltaic measurements. The dependence of the TAS spectra of DLC:Ag films and photovoltaic properties of DLC:Ag/Si heterostructures on the excitation wavelength was explained as a result of trapping of the photoexcited hot charge carriers in DLC matrix. The negative photovoltaic effect was observed for DLC:Ag/p-Si heterostructures and positive (“conventional”) for DLC:Ag/n-Si ones. It was explained by the excitation of hot plasmonic holes in the Ag nanoparticles embedded into DLC matrix. Some decrease of DLC:Ag/Si heterostructures photovoltage as well as photocurrent with DLC:Ag film thickness was observed, indicating role of the interface in the charge transfer process of photocarriers excited in Ag nanoparticles.

## Background

Nanostructures of IB group metals like Cu, Ag, and Au are interesting because of localized surface plasmon resonance (LSPR) effect [1]. One of the prospective application areas for the plasmonics and plasmonic nanomaterials is photovoltaics where plasmonic nanostructures and metamaterials are used to control an interaction between light and solar cell. The increased optical path of light in solar cell as well as local increase of the absorption coefficient in vicinity of the plasmonic nanoparticle can be achieved [1, 2]. Recently, it was found that LSPR can be used for direct control of electrical properties of the solar cell. Plasmonic nanostructures, being in contact with the semiconductor, can either inject electrons into the semiconductor or trap electrons from the semiconductor as a result of the free charge carrier generation after interaction with the photons of the appropriate energy [2]. Strong photovoltaic effect was observed for subbandgap photons of energy close to the LSPR energy of gold nanoparticles, when TiO_2_ substrate of the dye-sensitized (excitonic) solar cell (DSSC) was covered by non-percolating layer of Au nanoparticles [3]. Such effect in principle can be applied for inorganic semiconductor (free charge carrier) solar cells, too. Till now, this effect was mostly studied for TiO_2_ covered by Au or Ag nanoparticles as well as Ag core shell nanoparticles covered by TiO_2_. However, the use of the nanoparticles synthesized by different wet chemical routes is not beneficial from the practical application point of view, i.e., because of the complications integrating this process into semiconductor device fabrication technologies. As an alternative approach to the fabrication methods mentioned above, deposition of metal and dielectric (semiconductor) nanocomposites by reactive magnetron sputtering or related physical vapor deposition-based methods could be considered. In such a way, metal nanoparticles embedded into the semiconductor or dielectric matrix can be grown during the same deposition process. In addition, dielectric or semiconducting matrix of the nanocomposite film with tunable refractive index can be used for the additional control of the optical properties of the plasmonic nanomaterial. Moreover, it can be applied to protect nanoparticles from unwanted environmental effects such as oxidation of Ag nanoparticles. It should be mentioned that Ag nanoparticles as a plasmonic material have some advantages over Au nanoparticles due to higher intensity of the LSPR [4], lower optical losses [5], and larger solar energy conversion efficiencies [6]. However, silver nanoparticles are prone to rapid surface oxidation when exposed to ambient conditions [7]. Thus, use of the plasmonic nanocomposite would eliminate the main advantage of Au over Ag—its chemical inertness. However, in such a case, one should look for other types of dielectric matrix because mostly used TiO_2_ as an oxide has some disadvantage as a matrix material. It is not able to efficiently protect embedded Ag nanoparticles from the oxidation [8, 9]. On the other hand, there are only few studies on plasmonic photoelectron excitation and emission in the systems containing semiconductor (or dielectric) material other than TiO_2_. Particularly, research on photovoltaic properties of the Ag core shell nanoparticles covered by oxide dielectric SiO_2_ [10, 11] as well as semiconducting oxide Fe_2_O_3_ thin layer on the top or beneath Au nanoparticles should be mentioned [12].

As one of the alternatives of matrix for Ag nanoparticles, amorphous diamond-like carbon (DLC) could be considered. DLC is an amorphous allotrope of carbon consisting of the sp^2^ bonded carbon nanoclusters embedded into the sp^3^ bonded carbon matrix. DLC is well known for its properties like high hardness, low friction, and chemical inertness as well as biocompatibility [13–16]. Refractive index, optical transmittance in a broad optical spectrum, including visible light and near IR ranges, and electrical properties of DLC films can be varied in a wide range choosing the deposition conditions. DLC films are deposited at room temperature by using a wide variety of the plasma based vacuum deposition processes that are compatible with the semiconductor device technology. This leads to different fields of applications such as optical windows, magnetic storage disks, bar code scanners, various mechanics, car parts, solid lubricants, biomedical coatings and micro-electromechanical devices [13, 15, 17], piezoresistive sensors [18–20], microwave switches [21], and electrically active high-power device passivation films [22]. DLC matrix was proved as an excellent matrix for metal nanoparticles being able to prevent nanoparticles from chemical and mechanical hazards.

Considering photovoltaic properties of structures that include plasmonic nanoparticles, one should pay attention to the internal photoemission of the plasmonic hot electrons generated at the surface of the nanoparticles that is limited by hot electron recombination processes. Hot electron population usually decays due to the three main processes: thermalization of the electrons or in other words electron-electron scattering; electron-phonon interaction which describes hot (thermalized) electron energy transfer to the nanoparticle lattice; and finally, energy transfer from nanoparticle to the surrounding matrix. The process time scales are 10–100 fs, 0.1–5 ps, 1–100 ps, respectively [23–27]. These processes can become even more complex in the case of the nanoparticles embedded into the dielectric or semiconductor matrix.

This article aims at understanding of charge carrier generation, transfer and recombination processes in DLC films with embedded Ag nanoparticles (DLC:Ag nanocomposites), and role of these processes in the performance of photovoltaic properties of DLC:Ag/Si heterostructures. The analysis was performed by using transient absorption (TAS) spectroscopy and photovoltaic measurements. The unusual dependence of the TAS spectra of DLC:Ag films and photovoltaic properties of DLC:Ag/Si heterostructures on the excitation wavelength was found and explained.

## Methods

### Deposition of Thin Films and Heterostructures

In the present study, DLC:Ag films were deposited by direct current (DC) unbalanced magnetron sputtering of silver target. The diameter of magnetron was 3″. Polished polycrystalline alumina, monocrystalline silicon, and quartz substrates were used. Mixture of the hydrocarbons (acetylene) and argon gas was used in the reactive magnetron sputtering. In all experiments, substrate-target gap was set at 10 cm, magnetron target current was 0.1 A, base pressure was 5 ⋅ 10^− 4^ Pa and work pressure was (4 ± 1) 10^−1^ Pa. Samples were deposited on grounded substrates. Ar gas flux was 80 sccm and C_2_H_2_ gas flux was 7.8 sccm. As reported by our previous study [28], DLC:Ag films deposited using these conditions revealed the highest SERS signal intensity between all DLC films containing Ag investigated. Thus, strong plasmonic field can be supposed. According to our previous XPS measurement data, these films contained 22 at.% of Ag [19, 29] while average size of the Ag nanoclusters was ~10 nm [30]. More information on chemical composition and structure of the samples can be found in [19, 29].

In most experiments, thickness of the films was 50 nm. To study the influence of the film thickness on the photovoltaic properties of the samples, film thickness was changed in 10–150 nm range.

**Fig. 1:**
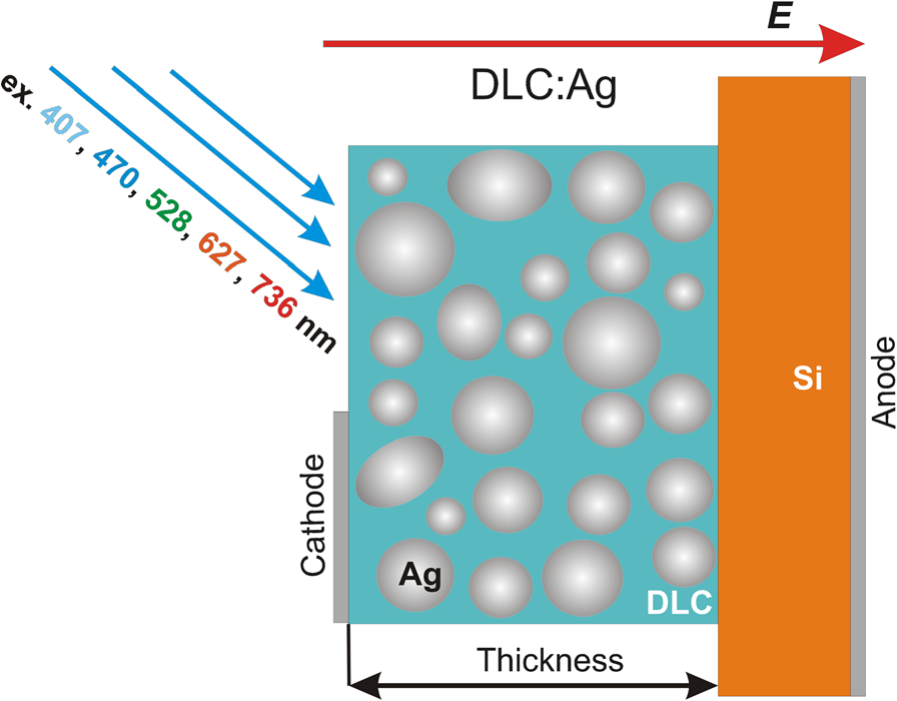
Schematic diagram of the sample used for studies of the photovoltaic properties of DLC:Ag/Si heterostructures

Fused silica was used as a substrate for studying of the optical properties. p and n type monocrystalline silicon was used as a substrate to study the photovoltaic properties of DLC:Ag/Si heterostructures. Vacuum-evaporated Al was used as electrode material (Fig. 1).

### Transient Absorption Measurements

The samples of DLC:Ag film on quartz were excited using ultrafast Yb:KGW laser Pharos (Light Conversion) with a regenerative amplifier generating 200 kHz repetition rate 290 fs duration pulses at 1030 nm wavelength. Avantes 2048 spectrophotometer was used for the steady-state absorption measurements. The proper excitation wavelength chosen from the highest absorption amplitude in the steady-state absorption spectra in most cases was tuned to 407, 470, 528, 627, and 736 nm with a collinear optical parametric generator Orpheus and harmonic generator Lyra (Light Conversion).

The samples of DLC:Ag film on quartz were probed with a white light supercontinuum generated using 2-mm-thick sapphire plate excited with the fundamental laser wavelength at 1030 nm. The spectral range of supercontinuum as well as the detection range of the TAS spectra dynamics was from 480 to 1000 nm. The excitation beam was focused to a spot of about 700 μm in diameter, while the probe white light supercontinuum beam diameter was of about 500 μm. Further details on the optical setup used can be found elsewhere [31].

OriginPro 2015 was used for the exponential and bi-exponential decay fitting of the TAS spectra.

### Photovoltaic Measurements

**Fig. 2:**
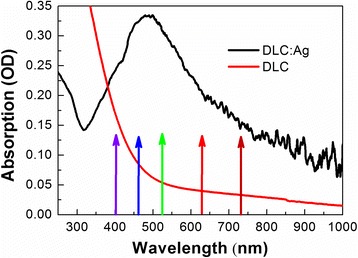
Steady-state absorption spectra of DLC:Ag nanocomposite film deposited on the fused silica substrate. The *arrows* at 407, 470, 528, 627, and 736 nm indicate excitation wavelengths used in the TAS spectroscopy and photoelectrical measurements

Photovoltaic properties of the DLC:Ag/Si heterostructures (Fig. 1) were investigated by using picoammeter Keithley 5487 and different light sources. In most experiments, light-emitting diodes (LED) were used as monochromatic sources of different wavelength light. Central LED emission wavelengths were very close to the ones used in the TAS measurements of DLC:Ag on quartz, i.e., 406, 470, 528, 627, and 736 nm (Fig. 2). In some cases, solar spectra simulator Scientech SF150B (Scientech Inc., Canada) was used as a broadband light source. Air Mass Filter AM1.5 spectra (300–1100 nm) was applied.

## Results

### Optical Properties of DLC:Ag Nanocomposites

#### Steady-State Optical Properties of DLC:Ag Nanocomposites

First of all, optical absorption spectra of DLC:Ag nanocomposite as well as DLC matrix used should be described; therefore, pure DLC and DLC:Ag steady-state absorption spectra are presented in Fig. 2. The absorption peak at about 470 nm is attributed to the LSPR absorption, while increase of the absorption at 300 nm and lower wavelengths is related to the absorption in DLC matrix [29]. One can see that absorption due to DLC matrix above 600 nm becomes negligible.

#### Dynamic Optical Properties of Pure DLC Matrix

**Fig. 3:**
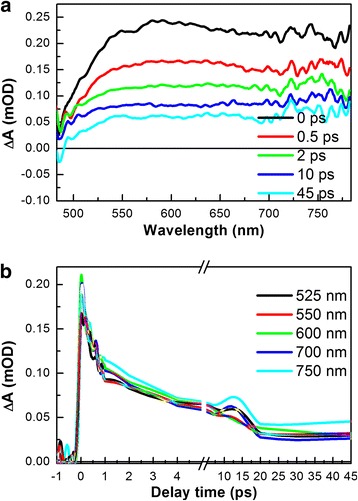
TAS spectra measured at different time of delay (**a**) and traces recorded at different wavelengths of TAS spectra (**b**) of neat DLC matrix. For the excitation of DLC matrix 7 μJ/cm^2^ intensity, 400 nm wavelength was used

In order to show how DLC interacts with plasmonic Ag nanoparticles in the case of DLC:Ag nanocomposites, first, the undoped hydrogenated DLC matrix (having no silver nanoparticles) was investigated by means of TAS spectroscopy (Fig. 3).

One can see that the TAS spectra of DLC matrix consist of positive signal in all measured spectral region (Fig. 3a). This signal can be attributed to the signal of induced absorption. In the region of the shortest wavelengths (below 500 nm), the positive signal decreases significantly showing that different processes take place which we can attribute to the ground-state bleaching [32].

Careful analysis of the TAS spectra dynamics (Fig. 3b) revealed that the curves of relaxation of pure DLC matrix for the all used excitation wavelengths consist of two components. Using bi-exponential decay function, the relaxation times of DLC matrix were revealed which are 0.41 ± 0.02 ps and 6.39 ± 0.45 ps, respectively, (decay kinetics at 600 nm was used for fitting because of the least noise and highest amplitude signal in this region).

#### Dynamic Optical Properties of DLC:Ag Nanocomposites

**Fig. 4:**
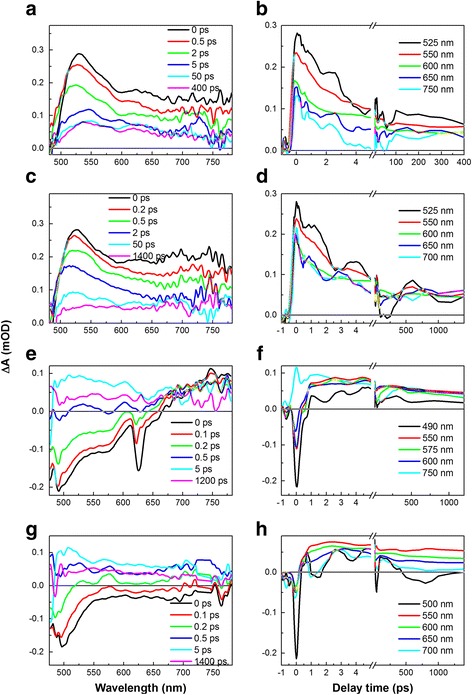
TAS spectra of DLC:Ag nanocomposites excited at various wavelengths: 406 nm (**a**, **b**), 470 nm (**c**, **d**), 627 nm (**e**, **f**), and 736 nm (**g**, **h**). The excitation intensities were 9 μJ/cm^2^, 11 μJ/cm^2^, 50 μJ/cm^2^, and 51 μJ/cm^2^, respectively

TAS spectra of the DLC:Ag nanocomposites were measured after excitation at 406 nm, 470 nm, 528 nm (not shown here because of very weak and noisy signal), 627 nm, and 736 nm (Fig. 4). The wavelengths of pump impulses represent the same wavelengths used for further photovoltaic measurements of DLC:Ag/Si heterostructures.

TAS spectra dynamics show clear dependence on the wavelength of excitation, e.g., DLC:Ag nanocomposites excited at 406, 470, and 528 nm show only positive signal in all spectra region, except dramatic amplitude decrease below 500 nm. The origin of this decrease may be similar to the pure DLC matrix.

Sample of DLC:Ag excited at higher wavelengths of 627 and 736 nm shows strong, short-living negative signal. The relaxation of this signal is much faster than that of the positive one. The lifetime of this signal suggests that it could be attributed to LSPR relaxation, and the process called electron-phonon interaction is responsible for this short decay [23].

Fitting of relaxation lifetime of the negative signal for the sample excited at 627 nm, using exponential decay function on TAS traces at 550 nm (we tried to avoid noises at 500 nm) provides a value of 0.36 ± 0.04 ps (Table 1). This value is similar to the results reported in [24]. On the other hand, it is at least one order faster than the ones declared in the references [33, 34]. These differences may occur because of different excitation intensities used in [33]. In our case, it was 50 μJ/cm^2^ that is similar to the intensity used in [24] (50–200 μJ/cm^2^). While in [33], much weaker excitation intensity was used (5 mJ/cm^2^).

**Table 1:** Lifetimes of TAS spectra relaxation (the kinetics at 600 nm probe wavelengths are presented because they demonstrate best SNR)

Sample	Excitation wavelength (nm)	Probe wavelength (nm)	*τ*_1_ (ps)	*τ*_2_ (ps)
DLC	400	600	0.41 ± 0.02	6.39 ± 0.45
DLC:Ag	406	600	0.75 ± 0.09	6.11 ± 0.60
	470	600	0.70 ± 0.06	7.36 ± 0.48
	627	550	0.36 ± 0.04	–
	736	500	0.22 ± 0.03	–

Lifetimes of the negative signal relaxation of DLC:Ag 22 at.% were explored for TAS data received under excitation at 627 and 736 nm

No phonon-phonon interaction was registered. Probably, TAS signal of DLC overruns this signal, which should have quite low intensity [34]. After less than 0.5 ps, the negative signal decays and transforms to the long-lived positive signal, which can be attributed to DLC matrix TAS signal. This approach allows to explain TAS signal of DLC:Ag after excitation at 627 and 736 nm, but unfortunately, it does not explain so well DLC:Ag relaxation signal after excitation at 406, 470, and 528 nm wavelengths, despite the fact that Ag nanoparticles in this spectral region should absorb light more intensively (Fig. 2).

The main peculiarity of these spectral data (406, 470, and 528 nm excitation) is an absence of the negative signal and high-relaxation lifetime that appears too long for the relaxation of plasmons. In fact, these TAS spectra are much more similar to the ones recorded for pure DLC matrix signal than to the LSPR of Ag nanoparticles relaxation dynamics. One could suppose that only DLC matrix is excited in this area, but the steady-state absorption spectra in Fig. 2 show different data (the intense absorption peak of DLC:Ag nanocomposites is at around 470 nm). While the absorption of DLC matrix and DLC:Ag nanocomposites is comparable at 406 nm, absorption by Ag nanoparticles at 470 nm should be larger than that by DLC matrix. Nevertheless, the TAS signal of DLC matrix completely overruns the signal of LSPR of Ag nanoparticles. This probably can be explained by interaction between the DLC matrix and Ag nanoparticles as charge transfer from Ag nanoparticles to DLC matrix, although it should be a very fast process because we were not able to distinguish it in the TAS relaxation dynamics.

We have also compared the relaxation decay times of DLC:Ag nanocomposites excited at 406 and 470 nm with the ones of pure DLC matrix. Fitting by the bi-exponential decay function shows that relaxation times of the “slow” relaxation component for DLC:Ag films (excited by 406 and 470 nm) are very similar to ones of the DLC matrix, showing that all these three TAS spectra represent the dynamics of DLC matrix (Table 1). Slow relaxation component disappears in the case of the DLC:Ag film TAS spectra excited by higher wavelength light (627 and 736 nm). Thus, it can be supposed that this component is related with processes in DLC matrix (Table 1). Relaxation dynamics of the positive signal consists of two components “fast” (*τ*_1_) and slow (*τ*_2_). The negative signal relaxation has only fast relaxation term in the negative area. That is why, only this component of relaxation time was explored. TAS signal changes fast from the negative to long-living positive signal. In order not to increase confusion, only relaxation time of short-living negative signal was included into Table 1.

### Photovoltaic Properties of DLC:Ag/Si Heterostructures

**Fig. 5:**
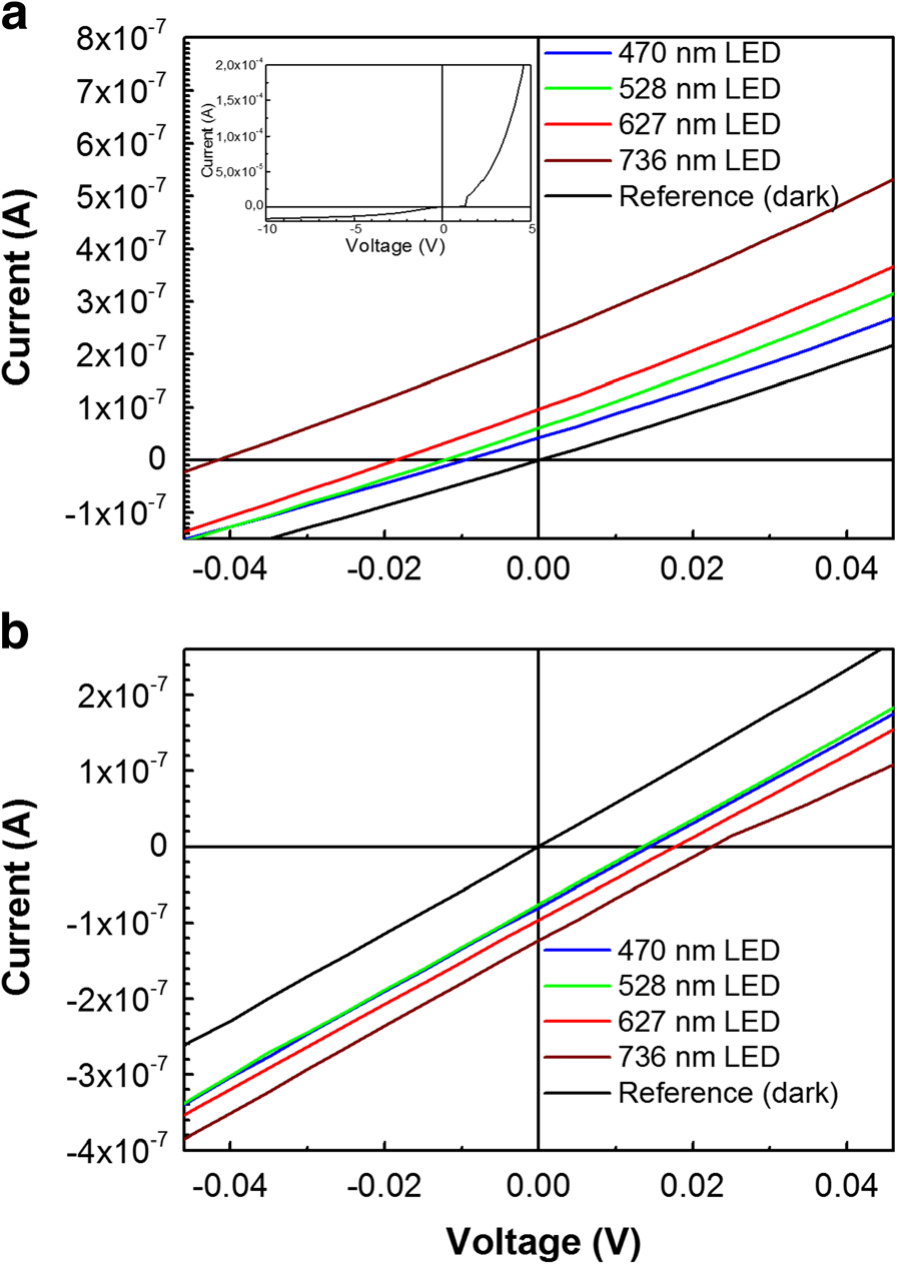
Typical I-U dependences of DLC:Ag/p-Si (**a**) and DLC:Ag/n-Si (**b**) heterojunctions illuminated with light of different wavelength. In the *inset* of Fig. 5a, dark I-V dependence of DLC:Ag/p-Si heterojunction in broader voltages range is presented

**Fig. 6:**
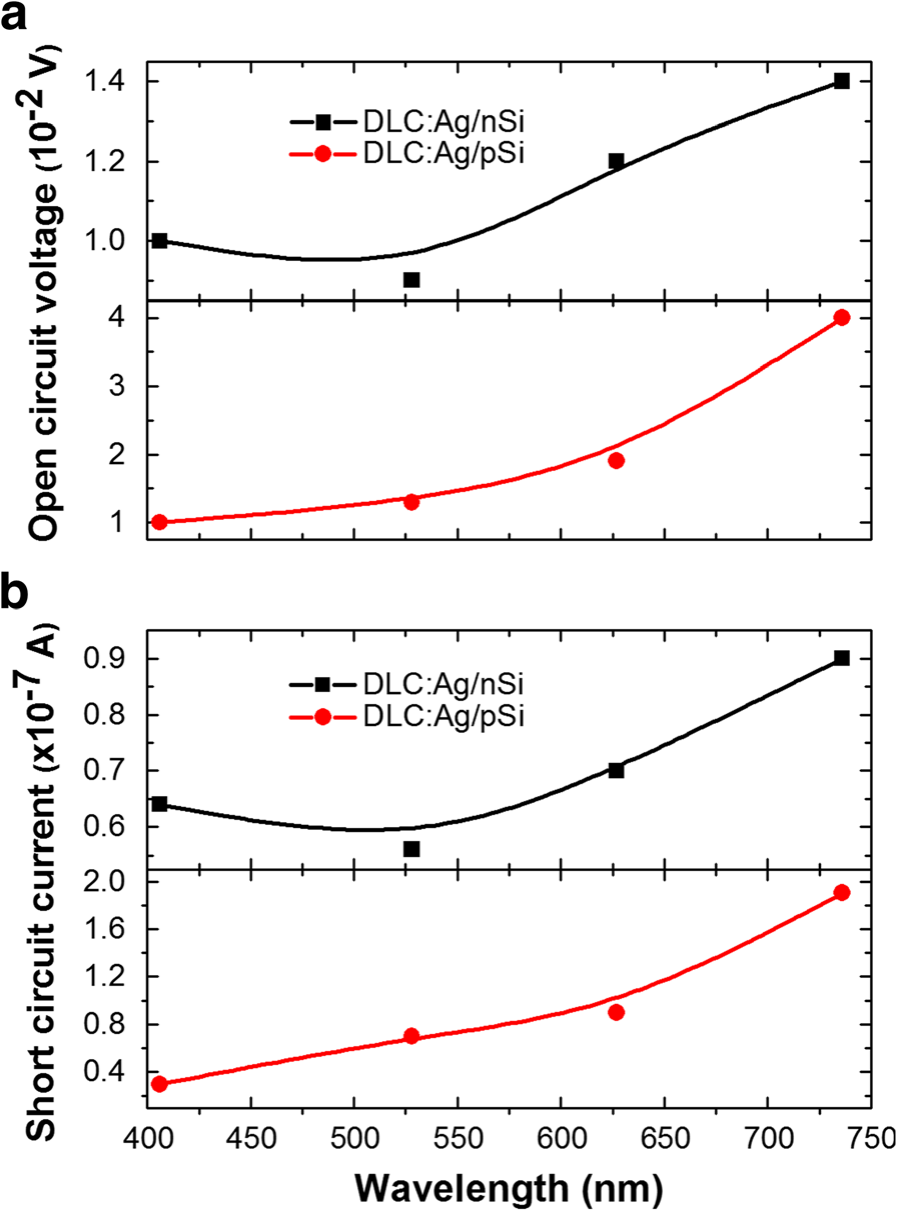
The dependence of the open circuit voltage (**a**) and short circuit current (**b**) on excitation wavelength for DLC:Ag/n-Si and DLC:Ag/p-Si heterostructures

Figures 5 and 6 illustrate photovoltaic properties of DLC:Ag/Si heterojunctions. It can be seen that in the case of the DLC:Ag/p-Si heterojunction, negative photovoltaic effect was observed (Fig. 5a).

According to Fig. 6a, b, open circuit voltage and short circuit current increase with the excitation wavelength. Such a behavior is a bit unexpected, as in numerous studies on generation of the plasmonic hot carriers, the conversion efficiency of incident photon to current was highest when the excitation wavelength was equal to the plasmonic absorbance peak wavelength [3, 10, 11, 35–41].

**Fig. 7:**
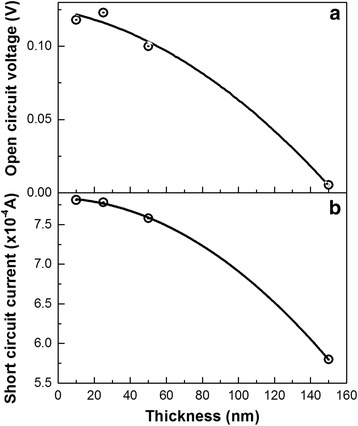
Open circuit voltage (**a**) and short circuit current (**b**) of DLC:Ag/p-Si heterojunction vs nanocomposite film thickness. Solar simulator with AM1.5 spectra was used for the photoexcitation

Afterwards, effects of the DLC:Ag film thickness on resultant photovoltaic properties of the herostructures were studied. Due to similar dependence of the photovoltaic parameters on the excitation wavelength for all the samples studied, effects of the DLC:Ag film thickness were investigated by using a solar spectrum simulator. In such a way, influence of the different wavelength photons was integrated by applying standardized light source. No clear dependence of the photovoltaic properties of DLC:Ag/p-Si heterojunctions on DLC:Ag film thickness in 10–50 nm range was found. Increase of the thickness up to 150 nm resulted even in some drop of the short circuit current and open circuit voltage (Fig. 7).

## Discusion

Analyzing photovoltaic properties of the studied heterostructures, one can see negative photovoltaic effect in the case of DLC:Ag/p-Si heterostructures. While for the DLC:Ag/n-Si heterostructures, positive (“conventional”) photovoltaic effect was registered. It should be mentioned that negative photovoltaic effect and negative open circuit voltage were reported in the case of some plasmonic nanostructures [42–44]. Particularly, visible plasmon irradiation of citrate coated Ag nanoparticles resulted in photogeneration of the negative photovoltage [42, 43]. It was explained by citrate photooxidation by Ag plasmon “hot holes” [43]. The negative photovoltage was observed for some ZnO:Al/SiO_2_/p-Si structures, when 1740–1925 nm wavelength light was used for excitation [44], while positive (conventional) photovoltaic effect was detected for 560 nm wavelength excitation. Thus, observed negative photovoltaic effect in DLC:Ag/p-Si heterostructure is mainly related to the DLC:Ag film. As for DLC/p-Si heterojunctions, only positive (conventional) photovoltaic effect was reported (see e.g., [45, 46]), one could suggest that some LSPR-related hot carrier generation processes took place in our experiment. Plasmonic photoexcitation of the hot holes can be supposed. This process should clearly prevail over excitation of the plasmonic hot electrons. It should be mentioned that according to the theoretical study presented in [47], in the case of Ag at some excitation wavelengths, only hot holes are collected and no electrons.

No clear dependence of the DLC:Ag/Si heterostructures photovoltage and photocurrent on DLC:Ag film thickness was found in 10–50 nm range, while meaningful decrease of photovoltage and photocurrent was observed for the DLC:Ag films with thickness of 150 nm. This effect was observed despite strong dependence of the absorbance of the DLC:Ag films on thickness. It seems that photoemission of the plasmonic charge carriers to the silicon takes place only from a very thin DLC:Ag layer that is close to the DLC:Ag/Si interface. Taking into account the data presented in Fig. 7 and discussions above, it can be supposed that in our case, average mean free path of the charge carriers photoemitted from Ag nanoparticles is clearly no more than the smallest thickness of the DLC:Ag films studied in this article, i.e., −10 nm. It should be mentioned that in [30], we have shown that the Ag nanoparticle size in DLC was 5–10 nm according to TEM, while according to the AFM data, most often, Ag nanoparticle size was ~10 nm. According to Fig. 4, the negative absorbance peaks, representing photogeneration of the plasmonic charge carriers, disappears in ~0.5 ps. Thus, it seems that due to the fast recombination of the photoelectrons, photoemission mostly takes place from the Ag nanoparticles, which are in close vicinity with the DLC:Ag/Si interface. However, taking into account the influence of the interaction between DLC matrix and Ag nanoparticles at lower excitation wavelength, it can be supposed that most of the photoelectrons reach silicon via ultrathin interlayer of DLC. Thus, it seems that recombination or trapping of the photoexcited charge carriers in DLC matrix takes place. Plasmonic photocarriers excited in Ag nanoparticles, which are located too far from the DLC:Ag/Si interface are unable to reach silicon. Decrease of the photovoltaic parameters with the increase of the DLC:Ag film thickness up to 50 nm can be explained by effects of the increased light absorption in the film. Less photons reach the DLC:Ag film zone near the interface with Si. Finally, it results in the decreased photocurrent and photovoltage.

Correlation between the optical absorbance spectra excited by different wavelength light and respective photovoltaic characteristics of the samples can be identified. Particularly, TAS spectra excited by 406–528 nm light are much more similar to the ones recorded for pure DLC matrix signal than to LSPR of Ag nanoparticles relaxation dynamics. Only in the case of the samples excited by 627 and 736 nm, the negative absorption signal, e.g., generation of the hot charge carriers can be seen. Such behavior was found despite that maximum of the plasmonic absorption peak was observed at ~500 nm. On the other hand, open circuit voltage and short circuit current of the samples clearly increased with the excitation wavelength. Such tendency was observed despite the decreased lifetime of the photoexcited charge carriers measured by TAS (see τ_1_ in Table 1). Thus, according to Table 1, decrease of the photovoltaic parameters with the excitation wavelength should be expected, as higher photoexcited charge carrier lifetime results in the increased short circuit current. Yet, in our case, the reverse tendency is observed. It seems that at lower excitation, wavelength influence of the DLC matrix results in suppression of the surface plasmon resonance-related hot charge carrier photogeneration. Alternatively, one can suppose that insertion of Ag nanoclusters into the DLC matrix results in the rearrangement of the density of the states (DOE). Particularly in [47], it was shown that in some cases, hot carrier collection efficiencies are higher in the case of 700 and 1500 nm excitation wavelengths in comparison with the higher energy 400 nm wavelength photons.

It should be mentioned that in numerous studies, hot charge carrier generation or photovoltaic quantum efficiency spectra correlate with the absorption spectra of the plasmonic absorber. In most mentioned studies, Ag or Au nanoparticles (nanostructures) were embedded into the wide bandgap semiconductor TiO_2_ or were at the interface with TiO_2_. However, it seems that quantum efficiency spectra of Au nanostructure/Si junctions follow this approach, too [48]. Only in [48], some similarities with the present study were observed, where deposition of Au nanoparticles on the magnetron-sputtered amorphous silicon film resulted in the increased photocurrent in 600–1000 nm range as well as increase of the photocurrent enhancement with photon wavelength in 450–1000 nm range with increased photon wavelength. No clear correlation between the changes of the photocurrent spectra and absorbance spectra of the plasmonic nanostructures was found in [48]. In [43], no correlation between the negative photovoltage excited in Ag nanoparticle and citrate system by different wavelength light and Ag nanoparticle optical absorption spectra was found, too. However, in [43], negative photovoltage increased with exciting photon energy. While in the present study, photovoltage decreased with the exciting photon energy.

The tendencies observed in this research can be explained by taking into account possible influence of the traps. The results reported in [49] should be mentioned as well. Negative photoconductivity was also observed for InAs nanowires [49]. Origin of the negative photoconductivity was attributed to the depletion of conduction channels by light-assisted hot-electron trapping [49]. Photoconductivity lowering was excitation wavelength dependent. Photocurrent decreased more when excitation wavelength was shorter (higher photon energy) [49]. In our case, we can suppose that at high excitation energies trapping of the hot charge carriers in DLC matrix or at the Ag nanoparticle/DLC interface takes place. Thus, photoexcitation of the plasmonic charge carriers competes with the trapping process. It results in suppression of the bleaching in DLC:Ag transient spectra excited by higher energy photons as well as decreased photocurrent and photovoltage. Energy of the hot charge carriers excited by longer wavelength photons is below trap-related states. In such a case, photoexcited plasmonic charge carriers avoid trapping. It results in the increased sample’s photocurrent and photovoltage at higher excitation wavelengths. This fact correlates with the TAS measurements when in TAS spectra of DLC:Ag films excited at higher wavelengths of 627 and 736 nm strong negative signal was observed, too.

We suppose that after excitation of the samples with shorter wavelengths (406–528 nm), charge transfer efficiency overruns hot electron relaxation. That is why we do not observe dynamics that we can attribute to LSPR relaxation, and DLC matrix signal dominates in the TAS spectra. While at longer wavelengths (627 and 736 nm), LSPR relaxation is much more effective process and we can see it clearly in the signal. From the first look, the photovoltaic characteristics should be better when excitation at shorter wavelengths is applied, but high-energy defects in the DLC matrix trap charges and reverse photovoltaic dependencies on the excitation wavelength completely. It is worth to mention that in some cases of plasmonic nanomaterials, photoexcitation of the charge carrier is more efficient at higher wavelengths. Particularly, under excitation at longer wavelengths (~700 nm), free electrons and holes can be generated in Ag nanoparticles while at shorter wavelengths (~400 nm), only generation of holes is effective [47].

## Conclusions

In conclusion, hot plasmonic holes were excited in Ag nanoparticles embedded into the DLC matrix. Therefore, negative photovoltaic effect was observed for DLC:Ag/p-Si heterostructures and conventional positive for DLC:Ag/n-Si ones. Some decrease of the DLC:Ag/Si heterostructures photovoltage and photocurrent with DLC:Ag film thickness was found in 10–150 nm range. It means that photocarriers excited in the Ag nanoparticles, which are located too far from the DLC:Ag/Si interface, are unable to reach silicon due to the recombination. The dependence of the TAS spectra of DLC:Ag films and photovoltaic properties of DLC:Ag/Si heterostructures on the excitation wavelength can be explained by trapping of the hot charge carriers in DLC matrix. This process prevailed over plasmonic photogeneration of the hot charge carriers at lower excitation wavelengths, while energy of the hot charge carrier’s photoexcited by longer wavelength photons was lower than the energy of the trap states. Therefore, these lower energy carriers avoided trapping by DLC defects.
